# A Preliminary Study toward a Rapid Assessment of Age-Related Behavioral Differences in Family Dogs

**DOI:** 10.3390/ani10071222

**Published:** 2020-07-17

**Authors:** Eniko Kubinyi, Ivaylo B. Iotchev

**Affiliations:** Department of Ethology, ELTE Eötvös Loránd University, 1117 Budapest, Hungary; ivaylo.iotchev@gmail.com

**Keywords:** ageing, cognition, social behavior, personality, excitability, dog

## Abstract

**Simple Summary:**

Cognitive aging in dogs has attracted interest due to their utility as an animal model for human aging and their need for veterinary care. The latter in particular would benefit substantially from standardized tests for fast and comfortable administration, which would reduce time and financial costs for both owners and practitioners. Here, we examine a battery of tests (the mini mental test, MMT) selected and adapted toward this end in a sample of 20 young (1–5y) and 26 old (10–15y) dogs. Each behavioral test was evaluated for its ability to discriminate between dogs based on their age category. Older dogs displayed less social interest, performed worse in a place-memory test, and seemed both less interested in and less fearful of a novel, moving object, aligning with previous findings and thus confirming the MMT’s external validity. The older dogs were also less excitable, assessed by a dog personality questionnaire. Based on these results, for future studies comparing the behavior of young adult and old dogs, we suggest four feasible subtests (greeting, problem box, memory, novel object) that can be conducted outdoors, without complex devices, in a short time (< than 15 min), and evaluated on the spot. To determine which performance levels are within the normal range for old dogs, larger samples grouped by breed and age will need to be tested in future studies.

**Abstract:**

Over the last few years, several efforts have been undertaken to characterize the aging process in dogs. In the present study, we evaluate a short protocol measuring dogs’ cognitive, social, and physical capacities. Our aim was to develop a feasible test battery, with minimal pre-training requirements, no complex devices, and which is set outdoors (i.e., a specific testing room is not needed). As ageing in dogs is usually associated with a decrease in activity, we also assessed the personality trait activity/excitability with a dog personality questionnaire. Four subtests proved sensitive to the dogs’ age. In particular, old dogs displayed less approaching and following behaviors toward an unknown but friendly human, showed both less avoidance and interest toward a novel object, looked less at the owner when faced with an unsolvable problem, and performed worse on the short-term memory task. Previous test procedures for investigating age-related changes involve expensive and/or complicated devices and extensive pre-training. The main advantage of the proposed battery is to reduce costs and efforts in veterinary assessments. Further tests in same-breed, large samples and between dogs with mild and severe cognitive impairments will be needed in order to further validate the battery.

## 1. Introduction

Among species studied to model the effects of healthy and pathological aging, the dog (*Canis familiaris*) occupies a unique niche [1]. The anthropogenic environment is the dog’s natural habitat [2], making it an ecologically valid model. Dogs display many humanlike social behaviors [3]. Finally, they are among the few model animals, alongside cats, that naturally develop dementia-like symptoms [4,5,6].

Aging naturally entails a decomposition of physical and mental capacities across most organisms, but species differences can be expected in the exact sequence in which functions begin to deteriorate, in the type of function, and in the speed at which function is lost. Great variation is also expressed within species. The majority of animal models do not show the rapid, overall loss of mental capacity that characterizes human dementia [1], and in humans, cats, and dogs, dementia conditions are considered pathological accelerations of the aging process. Over the last two decades, we have seen an increase in attempts to capture how age affects non-pathological canine behavior. Examples span from attention [7,8], memory [9,10], sleep physiology [11], personality [12], and social interaction [13] to higher cognitive functions, such as reasoning by exclusion and reversal learning [10,14,15]. A more practical problem related to the study of canine aging is how to standardize and optimize the behavioral testing protocols toward, for example, creating quick and easy assessment tools for practicing veterinarians.

Many of the test procedures published so far for cognitive aging in dogs could prove costly in terms of both time and finance if applied one on one in the veterinary practice. They involve either expensive and/or complicated devices, such as touch-screen computers [10] or other engineered testing apparatus [14]. Some tests investigating cognitive skills require extensive pre-training before the animals’ behavior can be evaluated with the test [10,15]. The administration of test batteries which capture spontaneous behavioral performances can save on both costs, although, like questionnaires, [16] they might not always distinguish clearly between behavioral differences caused by aging of the brain, body, and sensory systems, or be able to determine whether each system is affected [5]. For this reason, in some investigations, dogs were first pre-selected for equal performance based on sensory acuity tasks [15].

In this study, we present an initial evaluation of a short protocol measuring dogs’ cognitive, social, and physical capacities. The sub-tests are based on modified behavioral tests previously used toward this end: a battery for testing short-term visuo-spatial memory [9], a protocol used for measuring the spontaneous (social) activity and greeting behavior [17,18], and sensory acuity tests, used previously for pre-screening subjects [19]. Our aim was to develop a short, feasible test battery, later referred to as the mini mental test (MMT), that does not require pre-training or complex devices and can be conducted outdoors (i.e., it does not need a specific testing room). Evaluation criteria for each of the included sub-tests were their sensitivity to age effects on cognition and social interest, while task complexity and assessment duration were chosen by design for their ease of administration and quick testing. Since ageing in dogs is associated with a decrease in activity [12], we also assessed the personality trait activity/excitability with the Dog Personality Questionnaire (DPQ) [20].

## 2. Methods

### 2.1. Ethical Approval

The behavioral observations conducted in this study complied with national and EU legislation and institutional guidelines and were carried out according to Hungarian legislation (‘1998. évi XXVIII. Törvény’ 3. §/9.—The Animal Protection Act). The Hungarian “Animal Experiments Scientific and Ethical Committee” approved the experimental procedures under the numbers: PE/EA/2019-5/2017. Owners provided written consent for their dogs’ participation. The information included the owner’s right to withdraw their consent at any time. Owners could at any point decline to participate with their dog and could request that their data not be used and/or be deleted after collection. The study was performed following the recommendations in the International Society for Applied Ethology guidelines (https://www.applied-ethology.org/) for the use of animals in research. Non-invasive behavior tests are not considered animal experiments and are therefore allowed to be conducted without any special permission from the University Institutional Animal Care and Use Committee (UIACUC). The study was performed in strict accordance with the recommendations in the International Society for Applied Ethology guidelines for the use of animals in research.

### 2.2. Subjects

Young (N = 20, age range: 1–5 years, mean age: 3.1 ± 1.1 years, 30% males, 65% neutered, 7 mixed breed, 4 Australian shepherd dogs, and each 1 beagle, border collie, rough collie, English cocker spaniel, golden retriever, Hungarian greyhound, Siberian husky, Maltese, and standard poodle) and old (N = 26, age range: 10–15 years, mean age: 11.5 ± 1.2 years, 42% males, 84% neutered, 6 mixed breeds, 2 bichon Havanese, 2 Labrador retrievers, 2 standard poodles, 2 West Highland white terriers, 2 whippets, and 1 border collie, boxer, rough collie, German short-haired pointer, golden retriever, Hungarian vizsla, puli, shap-pei, wire-haired dachshound, and wire-haired fox-terrier) family (i.e., pet) dogs were recruited from volunteering owners for this study. Based on owners’ reports, the dogs did not suffer from major health problems; 89% were kept indoors or both indoors and outdoors, 43% were single dogs, 67% interacted (e.g., play, walk) with their owner for at least 1 h daily. We also screened them for auditory and visual perception and to check how valid these tests are in an outdoor setting (see Section 2.3.3. Sensory below). All dogs showed reactions to sounds in the sensory test, i.e., they reacted to the call of the experimenter. Two old dogs did not react noticeably in the vision test, but these animals were still otherwise able to perform in the tests, i.e., they found the food rewards and looked at the humans and the toy dog; therefore, we did not exclude them.

### 2.3. Mini Mental Test Protocol

The complete protocol consisted of eight sub-tests, which were all conducted outdoors: spontaneous activity (1), greeting (2), sensory (3), problem box (4), memory (5), bait track (6), toy dog (7), callback (8). Below, each sub-test is explained and described in detail. The behavioral variables coded for each subtest are presented in Table 1. The owner–dog pair and the experimenter met at the entrance of the university building and walked together for 40 m to the experimental area, where the owner read and signed the “Consent in Research Participation” form, while the experimenter set up the camera. Next, the experimenter showed the owner where to stand during the spontaneous activity test, stepped back behind the camera and started recording when the owner stood at the correct spot with the dog on a leash. The procedure before starting the video recording took approximately 5 min.

#### 2.3.1. Spontaneous Activity

The owner stood still silently and held the 1–1.5 m long leash of the dog. The time spent with locomotion was observed and coded for 30 sec.

#### 2.3.2. Greeting

**Figure 1:**
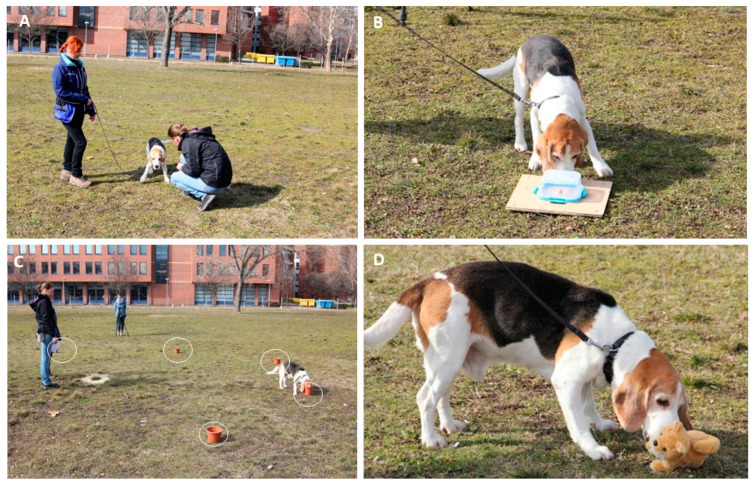
Example images from four sub-tests: greeting (**A**), problem box (**B**), memory (the pots are highlighted with yellow ovals) (**C**), novel object (toy dog, **D**). The persons identifiable in the images provided written consent for publication.

The experimenter approached the owner and the dog from the side in a non-threatening manner, stood out of leash (1 m), said “hi” to the dog, waited for 3 s, and observed whether the dog approached. If the dog approached, the experimenter came closer, pet the dog, and talked in a calm and friendly tone. If the dog did not approach, the experimenter switched to active encouragement by first calling, and then crouching. If the dog remained distant or fearful, the experimenter did not attempt direct contact and terminated the trial after 10 s. After greeting from a distance was completed, the experimenter took one step within the reach of the leash, still approaching from the side. The same greeting protocol was applied as above. At the end of the greeting sub-test, the experimenter collected the completed forms from the owner. The experimenter or owner (depending on the dog’s behavior during the greeting test) collected DNA samples from the animal (Figure 1A).

#### 2.3.3. Sensory

During this test, the dog was still leashed. Dog and owner stood still. The experimenter stood in front of the dog. With a piece of food in her left hand, she positioned the dog’s head, so that it was oriented ahead. With her right hand, she dropped the cotton ball on the side of the head (1x left side, 1x right side) in line with the animal’s eyes from 1 m height. Next, the experimenter stood behind the dog and made sounds with a ratchet and called the dog’s name, followed by the owner calling the dog’s name.

#### 2.3.4. Problem Box

A plastic, transparent kitchen box with a sealable lid was baited with a food reward (piece of sausage) to act as a problem box. After baiting, the experimenter stepped a meter away from the box and the animal was free to interact with it and eat the food. Phases: (i) Testing food motivation (once): the dog must learn that the box contains a treat: the experimenter puts food into the box while the dog is watching and the dog can eat the food, encouraged by the owner. The test is terminated if the dog does not eat the sausage; (ii) Two obtainable trials: E puts food in the box and covers it. The dog can obtain the reward by removing the lid; (iii) Blocked trial (30 sec): the baiting is the same but E locks the box, so it cannot be opened by the subject. Finally, the owner first leads the dog away from the box, and E then opens it and lets the dog eat the food (Figure 1B).

#### 2.3.5. Memory (Short-Term)

The starting position was marked with a blue buoy; around it, 5 pots (smeared with the bait controlling for scents) are placed in a semi-circle (Figure 1C). In each of the 5 trials, the dog’s attention was called to witness the hiding of a treat in one of the pots; the trial later concludes with a max. 1 min long recall test following a 30 s long distraction. A different pot was baited during each trial, never the same between trials, and the sequence of hiding positions was random. Before the dog’s recall of the hiding position was tested, it was turned away from the pots and talked to and petted (distraction). After 30 sec, the animal was returned to the pot arrangement for the next trial of the memory test. Each incorrect pot visited during the recall test was included in an error count.

#### 2.3.6. Bait Track

In this task, the dog was trained to follow the upside-down placed cup under which the experimenter hid a treat. The single training trial consisted of allowing the dog to eat the reward from underneath a yellow plastic cup placed 0.5 m from its starting position. If the dog refused to eat the food, the test was terminated. The testing included the use of two cups, of which one was baited and the other was empty. During testing, the difficulty was varied progressively in terms of how far the baited and empty cup changed positions relative to each other on an imagined circular trajectory: both cups moved by 90 degrees (1), 180 degrees (2), and completely exchanged their positions (3). The dog was tested once at each difficulty level. The dog saw the cups being moved and the behavior that was measured was whether the first chosen cup was the baited one.

#### 2.3.7. Novel Object (Toy Dog) 

During this test, the dog was left to interact for 30 s with an electronic, moving toy dog (Figure 1D), without interference from the experimenter or the owner. The test was initiated by the experimenter placing the toy (switched on) 0.5 m from the dog’s starting position. The test ended with the owner leading the dog away from the toy after 30 s. 

#### 2.3.8. Callback

The owner called the dog from a 30-m distance. The rewarding of the dog was not specified, i.e., the owner rewarded the dog as s/he usually did. This subtest was included as a control for physical problems not reported during pre-screening (observing the quality of locomotion) and generally to test responsiveness and obedience toward the owner.

### 2.4. Activity/Excitability Factor

We asked the owners to score how strongly they agreed with the 12 statements of the activity/excitability factor of the short form Dog Personality Questionnaire (DPQ) [20], (ranging from 1—I do not agree at all with the statement to 5—I fully agree), in Hungarian [12]. To calculate the facet and factor scores, we used the scoring key for the DPQ Short Form, provided by the author. The scores for each relevant raw item were averaged to create the facet scores. The factor scores were produced by averaging the scores of the facets that made up each specific factor. The factor was divided into facets: excitability, playfulness, active engagement, and companionability (3 items for each).

### 2.5. Variables and Statistical Analyses

As we aimed to develop a fast screening method, the behavior of the subjects was scored during testing. Behavioral measurements were scored in discrete levels (0–3 or 0–1) to limit the room for subjective interpretation between coders and to speed up coding (Table 1). The total number of correct first responses and (perseverance) errors were counted in the memory test.

These variables first underwent a frequency analysis, revealing that the sample was too uniform for further analysis in the case of several measurements (summarized in Table 1). In particular, if ≥70% of the sample showed the same behavior, the variable was excluded (see * in Table 1). 

We compared the proportions of scores between age categories with chi-square tests, and we compared the DPQ factor and facet score median differences between age cohorts with the Mann–Whitney U test. All analyses were performed in SPSS v25.

## 3. Results

Eleven behavioral variables were appropriate for score proportion comparisons in six sub-tests: *looking at owner* in the spontaneous activity, *approach* and *follow* of the experimenter in the greeting trial, *look at the owner/experimenter* in the inhibited trial of problem solving, *number of correct first choices, number of errors,* and *perseverative errors* in the memory trial, *correct choice* in trial 2 of the bait track test, *first retreat from toy dog* and *duration of looking at the toy dog* in the toy dog test. 

**Figure 2:**
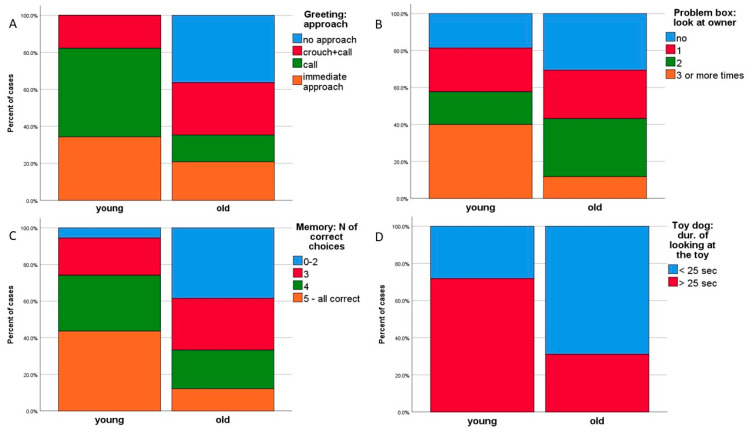
Proportions of subjects with age categories in four subtests.

Out of these variables, the proportions of old and young dogs differed in eight. More young than old dogs reacted to the experimenter during the greeting when she called them (60% of young and 31% of old dogs approached the experimenter upon calling (Figure 2A) and 70% vs. 31% followed her when she stepped away). Young dogs looked more often at the owner during the blocked trial of the problem-solving test (50% vs. 15%, Figure 2B). In the visuo-spatial memory test, 50% of young dogs chose the baited pot correctly at once, but only 15.5% of old dogs did so (Figure 2C). Concerning the total number of correct trials, 5% of young vs. 38.5% of old dogs made less than two instantly correct choices. Moreover, 70% young dogs vs. 35% old committed 0-1 errors during the memory test (i.e., they always visited the baited pot first or had only one error), while 30% vs. 65% visited at least two unbaited pots during the five trials before visiting the baited pots (the maximum number of errors was eight). A total 24% of the errors were perseverative errors (i.e., the subject chose the preceding trial’s baited pot during their first choice). More young than old dogs (75% vs. 42%) committed no perseverative errors. Fewer young dogs (20% vs. 50%) showed no signs of retreat when confronted with a novel object (a moving toy dog), and more young dogs (70% vs. 29%) looked at the object for a long time (nearly the total time of the test, Figure 2D, Table 1).

Owner-reported activity/excitability did not differ between the age cohorts considering the entire factor, but the excitability facet did (U = 147.00, df = 45, *p* = 0.02). Younger dogs were reported to be more excitable (young: median = 2.33, 95% CI = 2–3.33; old: median = 1.67, 95% CI = 1.67–2.00).

## 4. Discussion

The present study aimed to evaluate an easy-to-use protocol (MMT) for assessing the effects of aging on family dogs’ cognition, social behavior, and physical fitness. The MMT can be conducted outdoors, without complex devices, in a short time (<than 15 min), and evaluated real-time on the spot. The MMT was based in part on previously established testing procedures [9,17,18,19] and designed for fast and easy administration. The data obtained for this study allowed us to re-test some previously observed age-related changes in dogs which add to arguments for their replicability.

Following Bognár et al. (2020) [19], we evaluated the sensory capacities of dogs before enrolling them in our tests, in order to address sensory impairment as a possible confounding effect. Although two subjects did not pass the cotton ball test for visual acuity, they were still able to perform in the remaining sub-tests of this battery. Therefore, the cotton ball test is likely too conservative a criterion for exclusion or is compromised in an outdoor setting by additional factors like airflow. The results suggest that it is preferable to assess the sensory capacities in multiple scenarios to avoid false positives.

Only a subset of tests and variables used here proved sensitive to the dogs’ age. Old dogs displayed less approaching and following behaviors toward an unknown but friendly human, showed less avoidance and interest toward a novel toy dog, looked less at the owner when faced with an unsolvable problem, and performed worse on the short-term memory task. This implies that the present test battery can be shortened even further, to four sub-tests (greeting, problem box, memory, toy dog). Since the present sample, however, consisted of dogs without any owner-reported major health or cognitive problems (only one dog displayed signs of physical problems during the call-back test), it should be further investigated whether the sub-tests and/or variables not sensitive to age in a healthy sample could distinguish healthy and pathological aging in a more diverse sample. 

The lower rates of approaching and following behaviors in the old dog group toward the human experimenter observed here resemble previous findings of a lower number of approaches and social greetings toward conspecifics [13,21,22]. Fearfulness is not a likely explanation since older dogs were also less avoidant of the novel and moving toy dog in the toy dog sub-test, which reflects a loss in social interest or a more passive approach to social interactions [23].

The decline in memory performance with age was also previously observed in a version of the same task [9]. This supports the use of tests that measure spatial instead of object-oriented memory (e.g., Studzinski et al. 2006) [24]. The former is overall better developed in dogs [15]. In contrast, performance in the problem box sub-test (i.e., opening the box in the first two trials) was not sensitive to the animals’ age here, although other studies did find that cognitive abilities relevant in problem solving are affected by age [7,15]. Problem solving ability is influenced by not only cognition but also social factors, since in many problem-solving situations, dogs appear to resort to social strategies [25,26]. However, the decline in social interest with age [13,21] might help older dogs to focus on the problem.

The activity/excitability factor was not predictive of age in our sample, contrary to previous results with larger samples [12], save for one facet—excitability. Young dogs were reported to be more boisterous, less calm, and sought constant activity, indicating that these items are more predictive to age-related changes than other items of the activity/excitability factor.

A limitation of the present study is that each level of our categorical variables was represented by fairly small sub-samples (N < 30) from multiple breeds with different sizes and expected lifespans (i.e., ageing curves). Replicated results, like the association between age and correct responses in the memory test [9], suggest that despite the diverse breeds, the sample is not generally underpowered for testing associations with age, but the proportion comparisons reported here would need to be examined in the future with larger samples to determine the typical range of healthy ageing. Especially for the development of an instrument with clinical applicability, it will be crucial to re-examine what scores emerge in same-breed, large (e.g., N > 50) samples and between dogs with mild and severe cognitive impairments (as classified by veterinarian judgment, for example). Only then can clinical significance be assigned to specific scores (or score-ranges) obtained within and across the tests of this battery.

## 5. Conclusions

Social (and spatial) memory-related declines appear to be normal in healthy aging canines and more easily replicated than performance in more abstract tasks, when small samples and simple tests are used. In abstract problem solving, cognitive and social factors might interact, or these tests might alternatively be better for capturing pathological aging effects than studying healthy aging dogs. Older dogs appear to be less fearful/interested, based on a sub-test with a moving toy dog. These replications of previous observations also support the external validity of our proposed battery, which offers fast and comfortable administration, reducing time and financial costs for both owners and practitioners, for an easy assessment of age-related behavioral differences (and their degree). For example, the battery can be beneficial for shelter operators as behavior and age are critical factors in dog adoption, and the results can help to inform adopters in order to avoid dogs being returned to shelters. As a next necessary step, in order to create a battery suitable for a veterinary application, it is crucial to also compare healthy and pathologically aging animals in all of the sub-tests, as well as larger, same-breed samples, in order to achieve standardization.

## Figures and Tables

**Table 1:** Subtests, variables, definitions of scores, and proportions of subjects within age category (young/old). Variables lacking variation within a row (i.e., >70% of dogs show a single type of behavior) are marked with an asterisk (*). Significant young vs. old proportion differences are highlighted in bold (*p* < 0.05).

Sub-Test	Variable	Score 1	Score 2	Score 3	Score 4
1. Spontaneous activity	Activity *	<15 s (70/92)	>15 s (30/8)	-	-
	Vocalization *	No (90/88)	Yes (10/12)	-	-
	Laying down *	No (100/88)	Yes (0/12)		
	Looking at owner	0 (35/40)	Once (15/12)	Twice (25/20)	≥3 times (25/28)
2. Greeting	Approach experimenter	No approach (0/11)	Crouch + call (10/27)	**Call (60/31)**	Immediate approach (30/31)
	Follow experimenter	No approach (10/15)	Crouch + call (5/27)	**Call (70/31)**	Immediate approach (15/27)
3. Sensory	Turning head as a reaction to cotton ball * 1	No (0/12)	Yes (100/88)	-	-
	Turning head as a reaction to cotton ball * 2	No (0/8)	Yes (100/92)	-	-
	Reaction to a ratchet *	No (0/8)	Yes (100/92)	-	-
	Call by experimenter *	No (0/8)	Yes (100/92)	-	-
	Call by owner *	No (0/0)	Yes (100/100)	-	-
4. Problem box	Motivation for food *	No (0/0)	Yes (100/100)	-	-
	Obtained food 1 *	No (40/23)	Yes (60/77)	-	-
	Obtained food 2 *	No (15/8)	Yes (85/92)	-	-
	Blocked trial: looking at owner	0 (35/50)	Once (20/19)	Twice (15/23)	**≥3 times (30/8)**
	Blocked trial: looking at experimenter	0 (30/42.5)	Once (15/23)	Twice (20/15.5)	≥3 times (35/19)
	Blocked trial: orient toward problem box *	<50% (20/8)	>50% (80/92)	-	-
5. Memory	Number of correct first choices (0–5)	**0–2 (5/38.5)**	3 (15/23)	4 (30/23)	**5 (50/15.5)**
	Number of errors (unbaited pot visits) (0–8)	**0–1 (70/35)**	**2 or more (30/65)**		
	Number of perseverative errors only (0–2)	**0 (75/42)**	1 (20/35)	2 (5/23)	
6. Bait track	Correct choice 1 *	No (10/12)	Yes (90/88)	-	-
	Correct choice 2	No (50/40)	Yes (50/60)	-	-
	Correct choice 3 *	No (70/76)	Yes (30/24)	-	-
7. Novel object (toy dog)	First retreat from toy dog	>body length (35/12.5)	<body length (30/21)	Move head (15/16.5)	**No sign of retreat (20/50)**
	Approach toy dog *	<3 s (90/83)	>3 s (10/17)	-	-
	Touch toy dog *	No (80/67)	Yes (20/33)	-	-
	Duration of looking at the toy dog (sec)	**<25 s (30/71)**	**>25 s (70/29)**	-	-
	Vocalization *	No (90/79)	Yes (10/21)	-	-
8. Callback	Approach owner *	With delay (5/11)	Without noticeable delay (95/89)	-	-
	Physical problems *	No (100/96)	Yes (0/4)	-	-
	Motion type *	Trotting (0/12)	Galloping (100/88)	-	-
	Vocalization *	No (90/100)	Yes (10/0)	-	-
